# Reduction of Distortion Artifacts in Brain MRI Using a Field Map-based Correction Technique in Diffusion-weighted Imaging: A Prospective Study

**DOI:** 10.1007/s00062-023-01338-3

**Published:** 2023-08-28

**Authors:** Nils F. Grauhan, Natascha Grünebach, Lavinia Brockstedt, Antoine Sanner, Thorsten Feiweier, Vanessa Schöffling, Marc A. Brockmann, Ahmed E. Othman

**Affiliations:** 1 Department of Neuroradiology, University Medical Center Mainz, Langenbeckstraße 1, 55131 Mainz, Germany; 2 Technical University of Darmstadt, Darmstadt, Germany; 3 Siemens Healthcare GmbH, Erlangen, Germany

**Keywords:** Diffusion-weighted imaging, Field map correction, Geometric distortion, Stroke imaging, Image quality

## Abstract

**Purpose:**

The aim of this study was to evaluate the image quality and feasibility of a field map-based technique to correct for susceptibility-induced geometric distortions which are typical for diffusion EPI brain imaging.

**Methods:**

We prospectively included 52 patients during clinical routine in this single-center study. All scans were performed on a 3T MRI. Patients’ indications for MRI mainly consisted of suspected stroke due to the clinical presentation. For the morphological comparison of the corrected and uncorrected EPI diffusion, three experienced radiologists assessed the image quality of the sequences in a blinded and randomized fashion using a Likert scale (1 being poor; 5 being excellent). To ensure comparability of the two methods, an additional quantitative analysis of the apparent diffusion coefficient (ADC) was performed.

**Results:**

Corrected EPI diffusion was rated significantly superior in all the selected categories: overall level of artifacts (*p* < 0.001), degree of distortion at the frontal, temporal, occipital and brainstem levels (*p* < 0.001), conspicuousness of ischemic lesions (*p* < 0.001), image quality (*p* < 0.001), naturality (*p* < 0.001), contrast (*p* < 0.001), and diagnostic confidence (*p* < 0.001).

**Conclusion:**

Corrected EPI diffusion offers a significant reduction of geometric distortion in all evaluated brain regions and an improved conspicuousness of ischemic lesions. Image quality, overall artifacts, naturality, contrast and diagnostic confidence were also rated superior in comparison to uncorrected EPI diffusion.

## Introduction

Diffusion-weighted imaging has long been an integral part of MRI examinations in clinical routine [1]. In neuroimaging, it plays a crucial role in the early detection of ischemic infarction within the window of opportunity by visualizing early signal changes in the affected brain tissue mainly due to intracellular shift of water [2, 3].

A commonly used sequence for the acquisition of diffusion-weighted images is echo planar imaging (EPI). It enables rapid image acquisition, reducing signal loss and artifacts due to motion, but it is coupled with a higher sensitivity to susceptibility-related inhomogeneities in the main magnetic field [4]. This frequently causes geometric distortion of anatomical structures in the reconstructed images and thus a lack of agreement with undistorted high-resolution MRI scans, ultimately impairing diagnostic accuracy [5, 6].

A variety of methods have already been developed to compensate for the resulting loss in image quality, such as acquisition of multiple EPI images with different phase encoding directions [7] or using a registration template from undistorted sequences to estimate the extent of distortion [8]. In this work, we adopted the suggestion to acquire a separate field map which provides spatially resolved information of the absolute resonance frequency variation [9], and to use this information in a subsequent image correction.

Therefore, the hypothesis of our prospective study is that the application of corrected EPI diffusion is feasible in a clinical setting and will demonstrate a significant improvement in distortion artifacts, conspicuousness of ischemic lesions, naturality and contrast, the general image quality as well as the diagnostic confidence.

## Material and Methods

### Study Design

This prospective single center study was approved by the local ethics committee and conformed to the principles of the Declaration of Helsinki. All patients gave written informed consent. The acquisition period was between February 2022 and August 2022.

The following inclusion and exclusion criteria were defined: patients were included if suspicion of a stroke was raised by a referring physician, either in the emergency room or the hospital. They should be between 18 and 85 years of age, they were not allowed to have MRI contraindications as well as existing pregnancy or lactation and should not cause excessive motion artifacts. Patients were not included if they were not able to give informed consent.

### Image Acquisition

All measurements were performed on a 3T MAGNETOM Skyra (Siemens Healthcare, Erlangen, Germany) with a 64-channel head and neck coil. Corrected and uncorrected EPI diffusion data were obtained using research sequences acquired at the end of the patients’ clinically indicated examination. The relevant sequence parameters of the study sequences are listed in Table 1.

**Table 1:** Relevant MRI sequence parameters for corrected and uncorrected EPI diffusion

Parameter	Corrected EPI diffusion	Uncorrected EPI diffusion
Diffusion mode	N‑scan trace	3‑scan trace
b‑value 1 (averaging)	0 s/mm^2^, (3)	0 s/mm^2^, (3)
b‑value 2 (averaging)	1000 s/mm^2^, (3)	1000 s/mm^2^, (3)
Polarity	Bipolar	Bipolar
FOV (mm^2^)	230	230
TE	94 ms	94 ms
TR	5900 ms	5900 ms
Voxel size	1.2 × 1.2 × 3 mm	
Partial Fourier	6/8	6/8
TA	1.39 min	1.39 min

*FOV* field of view, *TE* echo time, *TR* repetition time, *TA* time of acquisition, *EPI* echo planar imaging

Field maps were acquired within 26 s using a triple-contrast gradient echo (GRE) sequence with the following parameters: field of view 320 × 320mm^2^; resolution 5.0 × 5.0 mm^2^; 58 slices (thickness 5 mm); echo times (TE) 2.4 ms, 4.6 ms, 7.1 ms; repetition time (TR) 10.9 ms. The orientation of the GRE scan was independent of the actual imaging scan, and position-matched, orientation-matched and resolution-matched field maps were interpolated directly on the scanner. Using an independent scan protocol for the field map enabled the same data to be reused for multiple subsequent imaging scans, without the need for repeated GRE acquisitions. Complex data from the three acquired contrasts were processed by the methods described in [10–12] to obtain absolute values of the local frequency offset. While the selected resolution of the field map limited the possibility to correct distortions on small spatial scales (e.g., in the temporal lobe), it allowed a robust and reliable estimation of actual absolute field variations.

Diffusion images were generated with a research application which combined a twice refocused spin-echo (TRSE) EPI acquisition with an integrated image correction algorithm. Using the spatially matched field maps, distortions of the diffusion-weighted magnitude images were corrected using interpolation and a Jacobian determinant-based density compensation as described in [13]. The apparent diffusion coefficient (ADC) maps were calculated based on diffusion-weighted images after distortion and density correction.

### Image Analysis: Morphological Comparison

The extent of distortion with a focus on the frontal and occipital pole of the brain as well as the temporal lobe and brainstem was of particular interest for our study and was rated using a 5-point Likert scale (1 being non-diagnostic area due to distortions, 5 being no distortions). Image quality evaluation comprised the assessment of the overall level of artifacts, overall image quality, diagnostic confidence and naturality (1 being poor, 5 being excellent). If ischemic lesions were present, their conspicuousness was rated using the same 5‑point Likert scale. The comparison was performed in a randomized, blinded manner by three radiologists (2 radiologists with 4 years and 1 radiologist with 9 years professional experience). All reviewers were given an introduction using two examples not included in the analysis. Images were viewed in 2 sessions separated by at least 4 weeks.

### Image Analysis: Comparison of ADC Values

Apparent diffusion coefficient (ADC) maps were automatically obtained from both corrected EPI diffusion and uncorrected EPI diffusion in all 52 patients. Measurements of ADC values were performed by placing a circular region of interest (ROI) of exactly 30 mm^2^ in 4 specific and corresponding regions of the provided ADC maps (left and right thalami as well as left and right precentral gyrus). Mean values in units of mm^2^/s were registered and saved to enable further statistical analysis.

### Statistical Analysis

For the statistical analysis we used R 4.1 (A Language and Environment for Statistical Computing (2008), Vienna, Austria). The non-parametric values of Likert scales are characterized with median and interquartile range (IQR). To determine whether corrected EPI diffusion scored significantly higher than uncorrected EPI diffusion, Wilcoxon signed-rank test was performed for all image quality criteria. Inter-rater reliability was determined using interclass coefficient (ICC) in an absolute-agreement one-way model. It was interpreted as poor (< 0.5), moderate (0.5–0.75), good (0.76–0.9) or excellent (> 0.9). To demonstrate the distribution of ADC values between corrected and uncorrected EPI diffusion in all 52 cases mean values and standard deviations were calculated separately for all four regions of interest and plotted using the Bland-Altman method (Fig. 4).

## Results

### Description of the Study Cohort

Corrected and uncorrected EPI diffusion images were successfully acquired in a total of 52 patients (28 male, 24 female) with a mean age of 59 years ranging between 21 and 85 years. In this cohort, a total of 18 patients revealed acute ischemic lesions, 7 patients with subacute ischemic lesions and a total of 4 patients with chronic infarction. Additional notable pathological findings included cerebral aneurysms, sinus thrombosis and cavernous hemangioma. A detailed description of the study cohort including indications for MR imaging is provided in Table 2.

**Table 2:** Study population

Patients, *n *(male/female)	52 (28/24)
Patient age in years, mean ± SD (range)	59 ± 17 (21–85)
*Patient diagnosis, n*	*Ischemic strokes in total, 28* multiple, acute, 11 singular, acute, 6 multiple, subacute, 4 singular, subacute, 3 multiple, old, 1 singular, old, 2 multiple, acute, and old, 1 *No pathological findings, 13* Aneurysms (with coiling/with clip/without intervention), 3 (1/1/1) Sinus thrombosis, 2 Leukoencephalopathy, 2 Microangiopathy, 2 Subdural hygroma, 1 Cavernous hemangioma, 1

*n* number, *SD* standard deviation

### Morphological Comparison

**Fig. 1:**
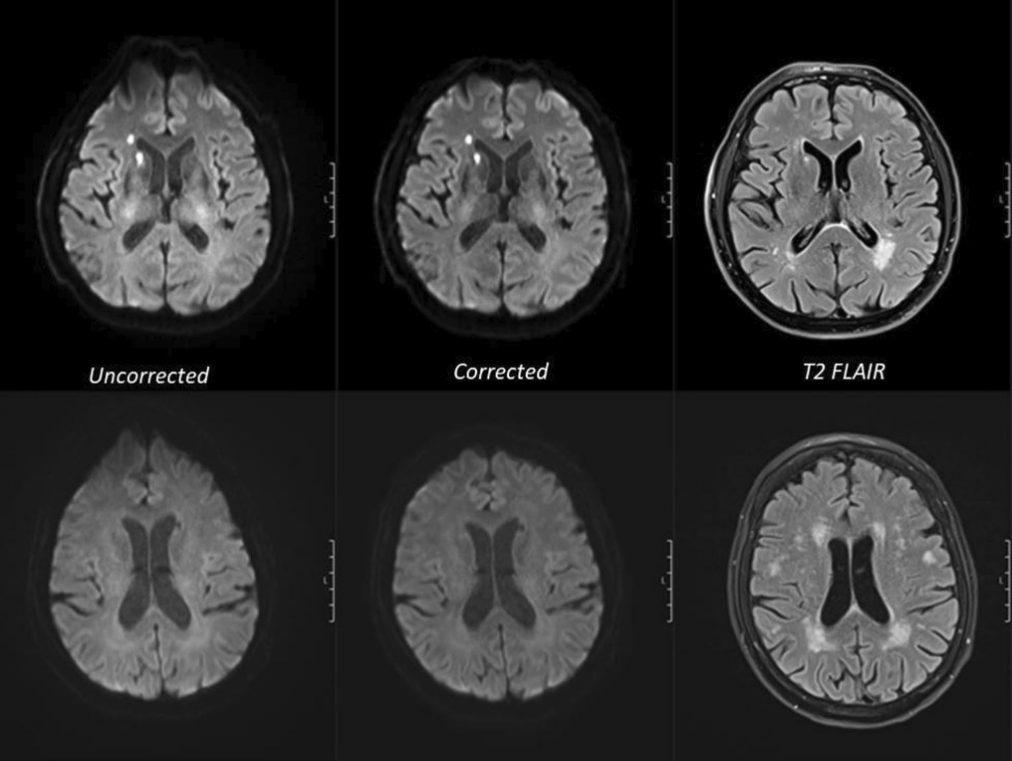
Examples axial MRI scans of two different patients (*upper and lower rows*): corrected EPI diffusion demonstrates a clear reduction of geometric distortion of the frontal lobe in both cases. Matched T2 FLAIR images are provided for comparison

**Fig. 2:**
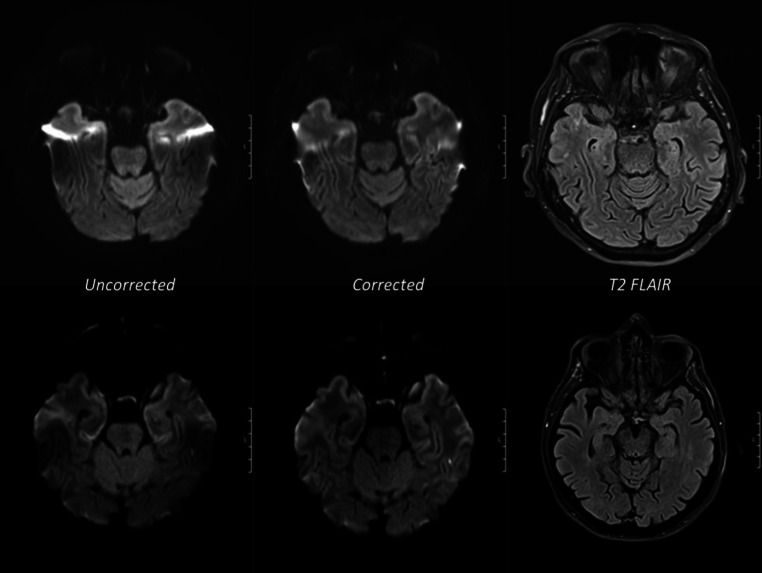
Examples of axial MRI scans of two different patients (*upper row*). Corrected EPI diffusion with a clear reduction in distortion artifacts affecting the temporal lobes. (*lower row*): corrected EPI diffusion showing improved conspicuousness of an ischemic lesion in the left temporal lobe. Matched T2 FLAIR images are provided for comparison

**Fig. 3:**
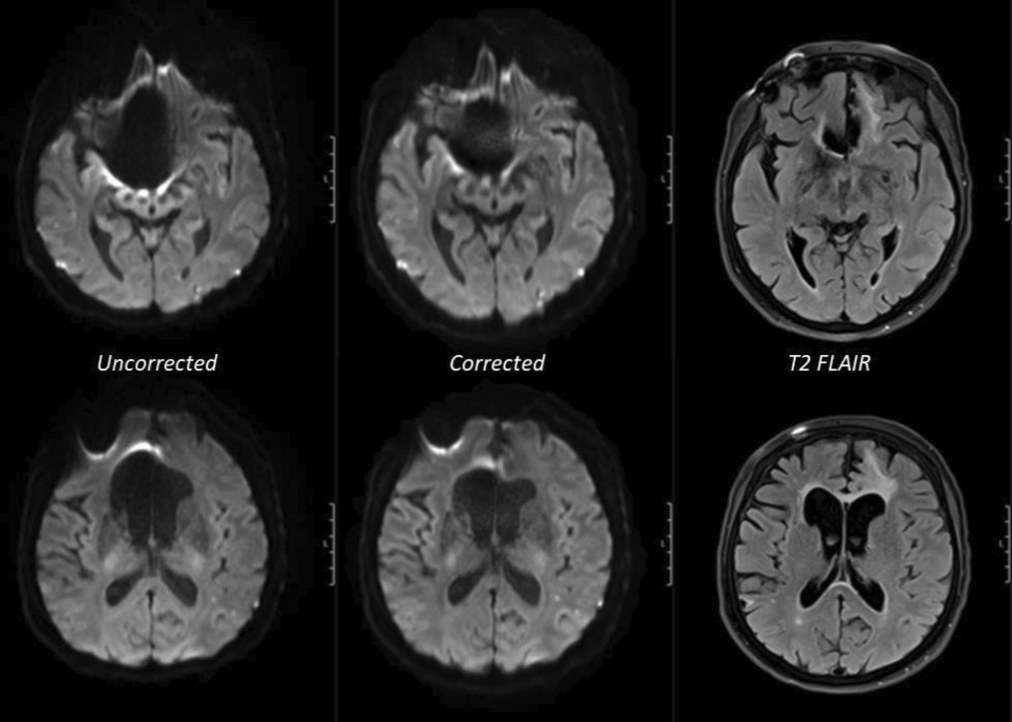
Examples of axial MRI scans of two different slice positions in the same patient with a surgically clipped aneurysm of the right anterior cerebral artery (*upper row*): substantially reduced artifact around the metal clip in corrected EPI diffusion. (*lower row*): consequentially improved geometry of the right anterior horn of the lateral ventricle due to the reduced metal-induced artefact. Matched T2 FLAIR images are provided for comparison

To demonstrate relevant differences between corrected and uncorrected EPI diffusion examples are provided in Figs. 1, 2 and 3. Among these images Fig. 2 clearly illustrates a reduction of signal pile up when using distortion correction. Wilcoxon-signed rank test demonstrated that corrected EPI diffusion was rated significantly better (*p* < 0.001) than uncorrected EPI diffusion in all aspects except for contrast in reader 1 and 3 (*p* = 0.014*; p* = 0.0017) and occipital distortion in reader 2 (*p* = 0.001). The overall median and interquartile range for corrected EPI diffusion were as follows: Image contrast 4 (3–4), naturality 3 (3–4), overall artifacts 3 (3–4), image quality 3 (3–4), ischemia conspicuity 4 (3–4), diagnostic confidence 4 (3–4), frontal distortions 4 (3–4), temporal distortions 4 (4–4), occipital distortions 4 (4–4) and brainstem distortions 4 (4–4). In contrast, median and interquartile range for uncorrected EPI distortions listed in the same order were as follows: image contrast 3 (3–3), naturality 3 (3–3), overall artifacts 3 (2–3) image quality 3 (3–3), ischemia conspicuity 3 (3–4) diagnostic confidence 3 (3–3), frontal distortions 2 (2–3), temporal distortions 3 (2–3), occipital distortions 3 (3–4) and brainstem distortions 3 (3–4). The majority of results demonstrated moderate interrater agreement (0.51–0.75). Agreement below 0.5 was present in image contrast (0.43). The medians with their interquartile ranges and interrater agreement are summarized in Table 3 for each image quality criterion.

**Table 3:** Results of image quality evaluation

	Reader 1				Reader 2			Reader 3			Overall		Agreement
	*Cor. Diff.* Median (IQR)	*Uncor.* *Diff.* Median (IQR)	*Wilcoxon-rank-sum test*	*Cor. Diff.* Median (IQR)	*Uncor.* *Diff.* Median (IQR)	*Wilcoxon-rank-sum test*	*Cor. Diff.* Median (IQR)	*Uncor.* *Diff.* Median (IQR)	*Wilcoxon-rank-sum test*	*Cor. Diff.* Median (IQR)	*Uncor.* *Diff.* Median (IQR)	*Wilcoxon-rank-sum test*	*ICC*
Naturality	3 (3–4)	3 (2–3)	*p* < 0.001	4 (3–4)	3 (3–3)	*p* < 0.001	4 (3–4)	3 (3–3)	*p* < 0.001	3 (3–4)	3 (3–3)	*p* < 0.001	0.53
Contrast	3 (3–4)	3 (2–4)	*p* = 0.014	4 (4–4)	3 (3–3)	*p* < 0.001	3 (3–4)	3 (3–3)	*p* = 0.017	4 (3–4)	3 (3–3)	*p* < 0.001	0.43
Artifacts	3 (3–4)	3 (2–3)	*p* < 0.001	3 (3–4)	3 (2–3)	*p* < 0.001	4 (3–4)	3 (3–3)	*p* < 0.001	3 (3–4)	3 (2–3)	*p* < 0.001	0.60
IQ	3 (3–4)	3 (2–3)	*p* < 0.001	4 (3–4)	3 (3–3)	*p* < 0.001	3 (3–4)	3 (3–3)	*p* < 0.001	3 (3–4)	3 (3–3)	*p* < 0.001	0.51
IC	4 (4–4)	3 (3–4)	*p* < 0.001	4 (4–4)	3 (3–4)	*p* < 0.001	4 (3–4)	3 (3–4)	*p* < 0.001	4 (3–4)	3 (3–4)	*p* < 0.001	0.57
DC	4 (3–4)	3 (3–3)	*p* < 0.001	4 (4–4)	3 (3–3)	*p* < 0.001	4 (3–4)	3 (3-3)	*p* < 0.001	4 (3–4)	3 (3–3)	*p* < 0.001	0.63
Frontal D	4 (3–4)	2 (2–3)	*p* < 0.001	3 (3–4)	2 (2–3)	*p* < 0.001	4 (3–4)	2 (2–3)	*p* < 0.001	4 (3–4)	2 (2–3)	*p* < 0.001	0.75
Temporal D	4 (3–4)	2 (2–3)	*p* < 0.001	4 (3–4)	2 (2–3)	*p* < 0.001	4 (4–4)	3 (3–3)	*p* < 0.001	4 (4–4)	3 (2–3)	*p* < 0.001	0.61
Occipital D	4 (4–4)	3 (3–4)	*p* < 0.001	4 (4–4)	3 (3–4)	*p* = 0.001	4 (4–4)	3 (3–4)	*p* < 0.001	4 (4–4)	3 (3–4)	*p* < 0.001	0.61
Brainstem D	4 (4–4)	3 (3–4)	*p* < 0.001	4 (4–4)	3 (3–4)	*p* < 0.001	4 (3–4)	3 (3–3)	*p* < 0.001	4 (4–4)	3 (3–4)	*p* < 0.001	0.69

*Cor. Diff.* corrected diffusion, *Uncor. Diff.* uncorrected diffusion, *IQ* image quality, *DC* diagnostic confidence, *IC* ischemia conspicuousness, *D* distortion, *IQR* interquartile range, *ICC* interclass coefficient

### Quantitative Comparison of ADC Values

**Fig. 4:**
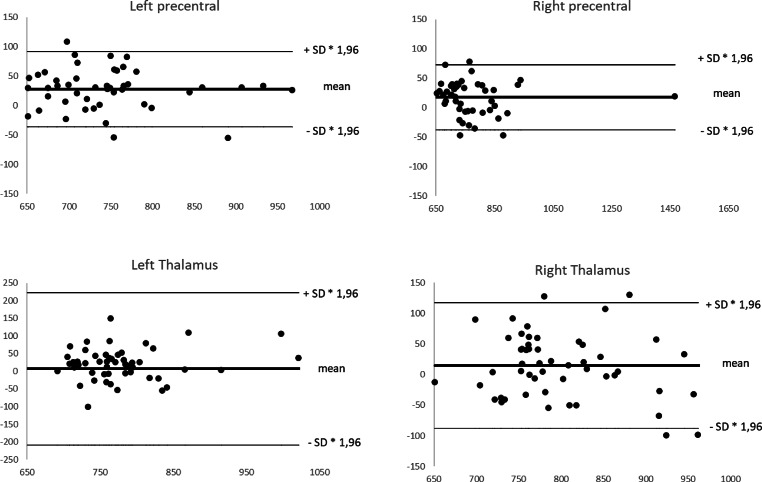
Bland-Altman plot with the distribution of ADC values measured in both corrected and uncorrected EPI diffusion each in four different regions of the brain (*left* and *right* precentral gyrus and left and right thalamus). Measurements are plotted in units of mm^2^/s using the average (*horizontal axis*) and the difference between two corresponding measurements (*vertical axis*)

The ADC values plotted separately for the four selected brain regions showed a distribution generally within the limits of agreement using a Bland-Altman plot as shown in Fig. 4. Three of the four analyzed regions (left and right precentral gyrus, right thalamus) included a maximum of four outliers.

## Discussion

EPI diffusion with field map-based correction was developed with the aim to decrease susceptibility-induced distortion and inhomogeneities typical for EPI-based diffusion imaging. The results demonstrate a clear improvement of image distortion, particularly at locations that tend to cause such severe artifacts regularly. These mainly include areas where bone, soft tissues or air interface and thereby can cause large differences in magnetic susceptibility [14, 15]. As a notable consequence, this can lead to a more difficult delineation or underestimation of ischemic changes in brain MRI [16–18]. Crucially, results of this study show that the conspicuousness of ischemic lesions improved significantly in the corrected EPI diffusion. Importantly, an additional review of our results revealed that no ischemic lesions were missed when viewing the corrected images. Otherwise, this could have indicated errors occurring in the field map used for image correction, which so far have not been reported in previous work using this method [13].

In addition to its influence on lesion detectability, geometric distortion in diffusion-weighted imaging can be problematic when pathological findings in distorted areas cannot be adequately associated with signal abnormalities in other sequences of a clinical MRI protocol [19, 20]. Specifically, matching diffusion-weighted images correctly with contrast enhanced sequences plays an important role in neuro-oncology, where correlation of sometimes very subtle changes can help to differentiate between malignancy or possible tissue necrosis after local radiation [21, 22]. Therefore, in a next step, the inclusion of additional MRI scans with a wider range of neurologic pathologies for which diffusion imaging is needed might be helpful to show whether the proposed correction method indeed offers diagnostic advantages in these cases as well.

Finally, overall artifacts, image quality, naturality and diagnostic confidence were rated superior in corrected EPI diffusion imaging. These results are in accordance with previous applications of the correction technique to improve the quality of prostate diffusion imaging [13]. We are therefore confident that the field map-corrected EPI diffusion is feasible in a clinical setting and can improve diagnosis in stroke imaging.

### Limitations

The study has some limitations. Small sample size limits the generalizability of the results. In addition, not all patients revealed ischemic lesions. Hence, inclusion of more cases with ischemic lesions in areas affected by distortion may further highlight the role of corrected EPI diffusion. Furthermore, consensus reading prior to subjective image evaluation consisted of only two examples, which may have impacted the inter-rater agreement with the majority of ICC values ranging between 0.51 and 0.75 and with 1 value below 0.5.

## Conclusion

Corrected EPI diffusion proved to be feasible in clinical routine MRI. Distortion artifacts were significantly reduced. Image quality, naturality, contrast, conspicuousness of ischemic lesions and diagnostic confidence were rated superior compared to uncorrected EPI diffusion.
